# National experience of alcohol septal ablation in patients with obstructive hypertrophic cardiomyopathy: A long-term multicenter retrospective study

**DOI:** 10.1016/j.ihj.2024.11.248

**Published:** 2024-11-22

**Authors:** Evgenii Shloido, Kirill Popov, Sergey Chernyshov, Maksim Kashtanov

**Affiliations:** a City Hospital No.2, Department of interventional cardiology, Saint-Petersburg, Russia; b Tyumen Cardiology Research Center, Tomsk National Research Medical Center, Russian. Academy of Science, Catheterization laboratory, Tomsk, Russia; c Tyumen Regional Hospital No.1, Department of Endovascular surgery, Tyumen, Russia; d Sverdlovsk Regional Hospital No.1, Department of Endovascular therapy, Yekaterinburg, Russia

**Keywords:** Alcohol septal ablation, Left ventricular outflow tract obstruction, Hypertrophic cardiomyopathy

## Abstract

**Objectives:**

Hypertrophic cardiomyopathy (HCM) is a widespread disease with an incidence of 1:200 in the general population and its surgical and interventional treatment is well-developed in western countries. This study is focusing on outcomes of HCM patients after alcohol septal ablation in Russian Federation.

**Methods:**

We conducted a multicenter registry to evaluate outcomes of obstructive hypertrophic cardiomyopathy (oHCM) patients after ASA.

Our study was focused on the following outcomes: (i) 30-day mortality, (ii) 30-day permanent pacemaker implantations, (iii) a residual obstruction occurrence, (iv) final maximal left ventricular outflow tract gradient, (v) long-term mortality, (vi) final heart failure functional class, (vii) freedom from sudden cardiac death. We conducted secondary analysis to assess outcomes in patients with single versus repeated ASA. The mean follow-up was 71 ± 47 months.

**Results:**

A total of 597 consecutive patients (54.9 % female) were enrolled in the Russian Alcohol Septal Reduction (RASA) registry from three interventional groups. The mean age was 56 ± 14 years. Thirty-day mortality rate was 0.7 % (4 patients). Permanent pacemakers were implanted in 42 (7 %) cases in 30-days follow-up. The resting LVOT gradient reduced from 64 ± 28 to 20 ± 13 mmHg (*p* < 0.0001), and the mean NYHA class decreased from 2.3 ± 0.7 to 1.3 ± 0.5 (*p* < 0.001). Long-term survival rates were as follows: 97.4 (95%CI: 96.2–98.7) %, 93.2 (95%CI: 91.0–95.3) %, 84.9 (95%CI: 80.7–89.4) % at 1-, 5-, 10-year follow-up, respectively. Patients after repeated ASA.

had similar long-term survival comparing to those who underwent single ASA (weighted log rank *p* value = 0.254). Heart failure class in the long-term and final gradient at the last follow-up were not statistically different between groups under study (*p* > 0.05).

**Conclusions:**

In our registry, alcohol septal ablation in patients with obstructive hypertrophic cardiomyopathy was safe in the short- and long-term follow-up. Outcomes of patients underwent repeated ASA were non-inferior to those after single ASA.

## Abrreviations

ASA: Alcohol septal ablation
oHCM: obstructive hypertrophic cardiomyopathy
TTE: transthoracic echocardiography
TEE: transesophageal echocardiography
CMR: cardiac magnetic resonance
RASA: Russian Alcohol Septal Ablation Registry
LVOT: left ventricular outflow tract
NYHA: New York Heart Association
IVS: interventricular septum
UFH: unfractionated heparin

## 1 Introduction

Alcohol septal ablation (ASA) is a well-established procedure for patients with obstructive hypertrophic cardiomyopathy (oHCM). The worldwide experience in such procedures demonstrates encouraging immediate and long-term results.^1^^,^^2^ However, the long-term influence of scarring due to ASA on outcomes is still being questioned. But recent data from the European registry showed durable long-term effects of the procedure and a reasonably low number of complications.^3^ According to recent American and European guidelines ASA may be considered as an alternative to surgical myectomy in oHCM patients with severe heart failure despite of optimal medical therapy in experienced centers.^4^^,^^5^ However, ASA procedure may be even preferred than myectomy in patients with older age, preexistent right bundle branch block and severe comorbidities.^6^

Recent study including more than 3500 HCM patients from 3 leading US centers demonstrated worse long-term survival after ASA compared to surgical myectomy.^7^ Anyway, the last meta-analysis of 27 studies (a total of 15 968 patients) didn't reveal a significant difference in long-term survival between these methods.^8^

The HCM population in Russia appears undertreated due to the lack of hospitals with structural HCM programs and experienced myectomy surgeons. In these settings, alcohol septal ablation may be a reasonable alternative to surgery. This paper presents the results of the Russian Alcohol Septal Ablation (RASA) registry, which currently includes data from three centers with almost 20 years of ASA experience.

## 2 Methods

### 2.1 The PICO question

(Patients)

We included patients with oHCM who underwent ASA between 2001 and 2022 in three leading Russian centers (hospitals from Saint-Petersburg, Yekaterinburg and Tyumen cities).

The diagnosis was based on typical clinical findings, electrocardiograms, and comprehensive imagine (transthoracic (TTE), transesophageal echocardiography (TEE), and/or cardiac magnetic resonance (CMR)).

Indications for the septal reduction were as follows: the presence of severe left ventricular outflow obstruction (≥50 mmHg) at rest or after provocation in cases when initial maximal medical therapy was not effective enough to reduce heart failure symptoms and/or to reduce left ventricular outflow tract (LVOT) gradient. Indications for ASA were supported by the local HCM team.

Patients with concomitant heart disease requiring open surgery (coronary artery bypass grafting, valve replacement, etc.) were excluded. ASA procedures were performed regardless of the presence or absence of mitral valve abnormalities, as well as the level of obstruction (LVOT, chordal, mid-ventricular).

### 2.2 Clinical follow-up was performed by office visit, phone contact, or structured follow-up

(Intervention)

All patients underwent a classic ASA technique with echo-contrast guidance.^9^ Procedures were performed under local anesthesia with analgosedation (fentanyl) during ethanol infusion. One or two arterial accesses were used to perform ASA and to evaluate hemodynamics. The electrode for the temporary pacing was routinely placed in the right ventricular apex for 3–5 days depending on the AV conduction. During ASA we used a standard unfractionated heparin (UFH) dose of 70–100 IU/kg. Dual antiplatelet therapy was used in cases of concomitant ASA and coronary stenting.

We used 6–7F guiding catheters to engage left main coronary ostium. Multipurpose or pigtail catheter was placed in the left ventricle close to apex. Hemodynamic tracings were obtained from two sites: left ventricle (pigtail, MPA catheters) and aorta (a tip of guiding catheter). To assess a severity of the LVOT obstruction we measured peak-to-peak gradient. The LVOT gradient exceeding 50 mmHg were considered significant. We used hydrophilic coronary wires to rich a target septal branch. Coronary over-the-wire (OTW) balloon (1.5–2.5 mm) was placed a in proximal part of target septal branch and inflated up to 6–8 atm.

To assess a perfusion zone of target septal branch we performed intraoperative transthoracic echocardiography. In order to more precisely define the perfusion zone of perforators, 0.5–2 mL of echocardiographic contrast agent was administered through the OTW balloon shaft. The contrast agents utilized included Sonovue (Bracco), agitated saline, and agitated Gelofusin 4 %(B.Braun). If the perfusion zone of target septal branch filled the septal muscular bulge in basal segments and matched with the level of obstruction we considered to infuse an ethanol. Desiccated alcohol 96 % (0.5–3 ml) was used. Predominantly three experienced ASA operators performed these procedures. There was no clear consensus regarding ethanol dosing; three major rules were used: (i) 1 ml per 1 cm of septum; (ii) fixed 3 ml dose, (iii) low-dose strategy depending on the LVOT gradient severity (resting PG < 50 mmHg - 0.5 ml, resting PG 50–100 mmHg – 1 ml, resting PG > 100 mmHg–1.5 ml). The mean dose of 96 % ethanol was 1.6 ± 0.9 ml. The next step was an inflated balloon exposure for 5–10 min following by the balloon deflation. After balloon deflation a final coronary angiogram was performed to confirm the perforator's occlusion and a patency of the rest coronary vasculature. Patients were transferred to intensive care unit.

(Comparison: study design)

This is an observational study that demonstrates current practice, procedural effects, and its results on mid- and long-term survival.

(Definitions)

• A criterion of residual obstruction was maximal PG ≥ 50 mmHg after provocation. Residual obstruction was assessed during the last check-up.
• Reoperation was defined as repeated ASA or mitral replacement and/or surgical myectomy.
• The septal reduction was defined as a difference of interventricular septum (IVS) thickness at baseline minus IVS thickness at the last check-up.
• The “mid-term” defined as a period between first month and 1 year of follow-up.
• The “long-term” meant the period of time exceeding 1 year of follow-up.

(Outcomes)

We assessed the following outcomes.

(i) 30-day mortality, (ii) 30-day permanent pacemaker implantations, (iii) a residual obstruction occurrence, (iv) final maximal left ventricular outflow tract gradient, (v) long-term mortality, (vi) final heart failure functional class, (vii) freedom from sudden cardiac death.

We conducted secondary analysis to assess outcomes in groups of oHCM patients after single versus repeated ASA (supplementary material). The following outcomes were evaluated.

(i) 30-day mortality, (ii) 30-day permanent pacemaker implantations, (iii) a residual obstruction occurrence, (iv) final maximal left ventricular outflow tract gradient, (v) long-term mortality, (vi) final heart failure functional class, (vii) myectomy cases after ASA sessions.

### 2.3 Data collection

Data were obtained from local databases and through direct calls to patients or relatives, or their family physicians. The Mandatory Health Insurance Fund kindly provided additional relevant information relating to the long-term survival rates for each patient (as well as the dates and causes of death).

### 2.4 Statistical analysis

We used R 4.4.1 package (Vienna) to analyze our data. Multiple imputation was used to deal with missing values via mice package and the “predictive mean matching” algorithm. The Shapiro–Wilk test was used to test continuous variables for normality. Continuous data have been presented as a mean ± standard deviation for variables with normal distribution and median (25th–75th percentile) for variables with non-normal distribution. Categorical data have been described as absolute numbers and relative frequencies. Non-categorical variables have been summarized using means and were compared using ANOVA or Kruskal–Wallis tests according to normality tests. The Kaplan–Meier method was applied to construct survival curves. The survival was presented with a 95 % confidence interval. For secondary analysis we adjusted initial imbalance between groups using the inverse probability of treatment weighting (IPTW) with stabilized weights. We used generalized linear modeling to calculate propensity scores and weights. We measured the following estimand (causal effect): an average treatment effect on the treated (ATT). A standardized mean difference >0.1 was considered significant. Weighted Cox proportional hazard model to obtain pooled hazard ratios with robust 95 % confidence intervals. A *p*-value <0.05 was considered statistically significant.

## 3 Ethical statement

The authors are accountable for all aspects of the work in ensuring that questions related to the accuracy or integrity of any part of the work are appropriately investigated and resolved. The study was conducted in accordance with the Declaration of Helsinki (as revised in 2013). The institutional review board of Sverdlovsk Regional Hospital No.1 approved the post hoc analysis of the data for this study (No. 40/2023) and patients’ informed consent was waived due to the retrospective nature of the study.

## 4 Results

**Fig. 1:**
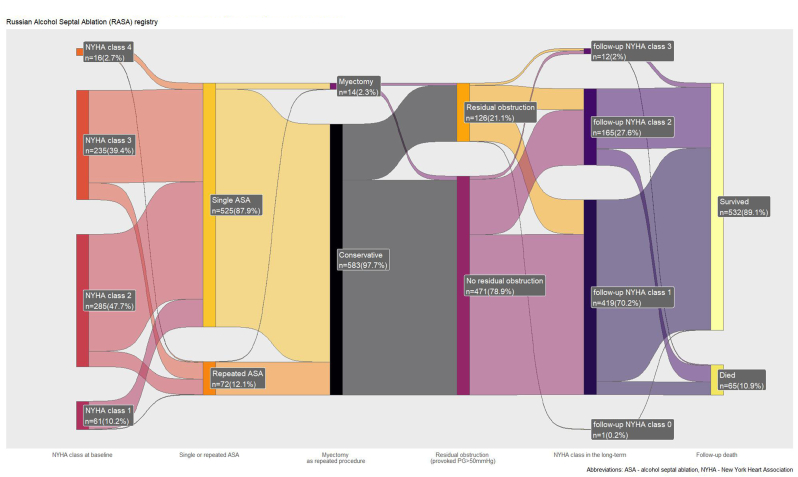
Sankey diagram describing study's flowchart.

A total of 597 HCM patients were included in our study. The median age was 56 (48–66) years. Female patients account for 54.9 % (328 patients). All relevant clinical characteristics are presented in Table 1. Study's flowchart presented via “Sankey” graph in a Fig. 1.

**Table 1:** Demographic characteristics, (*n* = 597).

Variable	Value
female	328 (54.9 %)
BMI, kg/m^2^	29.6 (26.4–33.0)
age	56 (48–66)
Family history of SCD	87 (14.6 %)
Syncopes	196 (32.8 %)
nsVT	29 (4.9 %)
PM or ICD	10 (1.7 %)
Coronary artery disease	87 (14.6 %)
NYHA III-IV class	251 (42.1 %)
Smoking	141 (23.6 %)
DM	58 (9.7 %)
Arterial hypertension	405 (67.8 %)
Atrial fibrillation	123 (20.6 %)
SCD Risk score	3.4 (2.3–5.3)

BMI - body mass index, SCD - sudden cardiac death, PM - pacemaker, ICD - implanted cardioverter-defibrillator, DM - diabetes mellitus.

nsVT – non-sustain ventricular tachycardia.

### 4.1 Short-term results

Thirty-day mortality was 0.7 % (4 patients). Permanent pacemakers were implanted for 7 % (42) patients during 30 days of follow-up. Causes of death were as follows: sudden death due to electrode dislocation or its dysfunction – 3; sepsis – 1.

### 4.2 Procedural effects

We observed a gradual decrease of IVS thickness during a period of follow-up from 21.2 ± 3.0 mm (baseline) up to 16.8 ± 3.2 mm (the last visit) (*p* < 0.001). The mean septal reduction was 4.4 ± 2.8 mm (median 4 mm).

**Fig. 2:**
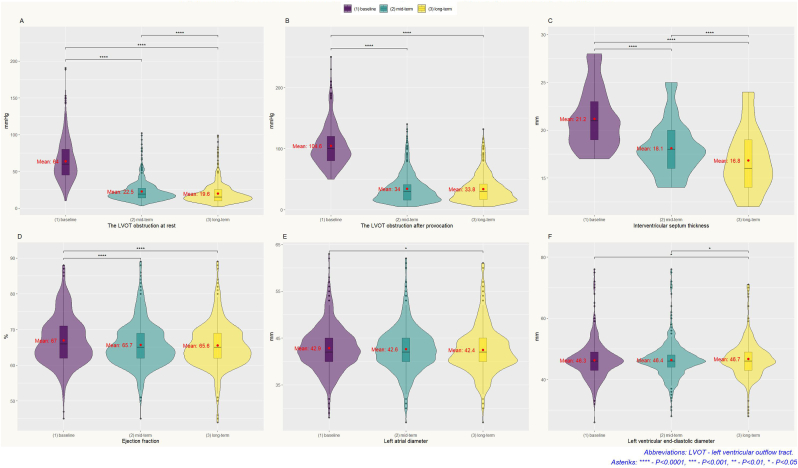
Long-term effects of alcohol septal ablation in obstructive hypertrophic cardiomyopathy patients.

After ASA the maximal resting LVOT gradient decreased from 64 ± 28 mmHg to 22 ± 13 mmHg in the mid-term and then to 20 ± 13 mmHg in the long-term follow-up (*p* < 0.001). The maximal provoked LVOT gradient decreased from 105 ± 34 mmHg to 34 ± 23 mmHg in the mid-term and then to 34 ± 22 mmHg in the long-term follow-up (*p* < 0.001). Left atrium diameter in the long-term follow-up was lower comparing to the initial size (42.9 ± 4.7 mm vs 42.4 ± 5.0 mm, *p* < 0.05). Left ventricular ejection fraction decreased from 67 ± 7 % at baseline to 66 ± 6 % in the mid-term (*p* < 0.0001), and then stayed similar during the next follow-up (66 ± 6 % in the long-term, *p* > 0.05). Left ventricular end-diastolic diameter in the mid-term was not statistically different compared to baseline (46.3 ± 6.0 vs 46.4 ± 5.7, *p* > 0.05), but in the follow-up it was statistically larger than a baseline diameter (46.7 ± 5.7 vs 46.3 ± 6.0, *p* < 0.05). All findings are summarized in Table 2 and Fig. 2.

**Table 2:** Effects of alcohol septal ablation on echocardiographic measures.

Variable	(1) baseline^a^, *n* = 597	(2) mid-term^a^, *n* = 597	(3) long-term^a^, *n* = 597	*p*-value^b^	(1) vs (2)	(1) vs (3)	(2) vs (3)
**The peak LVOT gradient at rest, mmHg**	64 (28)	22 (13)	20 (13)	**<0.001**	∗∗∗∗	∗∗∗∗	∗∗∗∗
**The peak LVOT gradient after provocation, mmHg**	105 (34)	34 (23)	34 (22)	**<0.001**	∗∗∗∗	∗∗∗∗	ns
**Interventricular septal thickness, mm**	21.2 (3.0)	18.1 (2.7)	16.8 (3.2)	**<0.001**	∗∗∗∗	∗∗∗∗	∗∗∗∗
**Ejection fraction, %**	67 (7)	66 (6)	66 (6)	**<0.001**	∗∗∗∗	∗∗∗∗	ns
**Left atrium diameter, mm**	42.9 (4.7)	42.6 (4.8)	42.4 (5.0)	0.045	ns	∗	ns
**Left ventricular end-diastolic diameter, mm**	46.3 (6.0)	46.4 (5.7)	46.7 (5.7)	0.002	ns	∗	∗

Abbreviations: LVOT - left ventricular outflow tract, ns – non-significant difference.

Asteriks: ∗∗∗∗ - *p* < 0.0001, ∗∗∗ - *Р*<0.001, ∗∗ - *p* < 0.01, ∗ - *p* < 0.05.

a Mean (SD).

b Kruskal–Wallis rank sum test.

### 4.3 Complications

All perioperative and 30-day complications are listed in Table 3. We presented an electrode dislocation as a complication because it seems a common (14 patients, 2.3 %) and potentially dangerous event in these settings.

**Table 3:** 30-days complications.

No.	Complication	Number of cases (%)
1	*Permanent pacemaker implantation*	42 (7 %)
2	*Hematoma (after arterial access)*	30 (5 %)
3	*Electrode dislocation*	14 (2.3 %)
4	*Deep vein thrombosis*	2 (0.33 %)
5	*Femoral pseudoaneurysm*	2 (0.33 %)
6	*Hospital pneumonia*	1 (0.17 %)
7	*Ventricular fibrillation*	1 (0.17 %)
8	*Gastrointestinal bleeding*	1 (0.17 %)
9	*Left main dissection*	1 (0.17 %)
10	*Coronary artery thrombosis*	1 (0.17 %)
11	*Non-target ablation*	1 (0.17 %)

### 4.4 Long-term results

The mean follow-up was 70 ± 47 (a median of 62) months (2985 patient-years). During follow-up 65 patient died, corresponding to all-cause mortality rate of 2.2 deaths during 100 patient-years.

Long-term survival was as follows: 97.4 (95%CI: 96.2–98.7) %, 93.2 (95%CI: 91.0–95.3) %, 84.9 (95%CI: 80.7–89.4) % at 1-, 5- and 10-year follow-up.

**Fig. 3:**
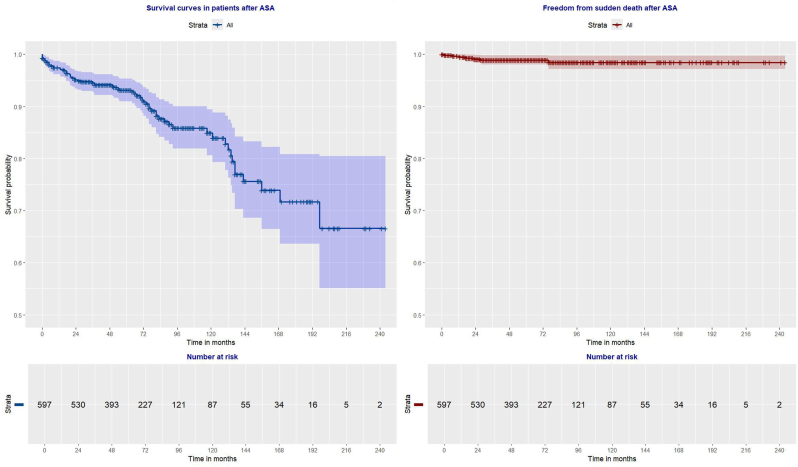
Survival curves in HCM patients after alcohol septal ablation.

Freedom from sudden cardiac death in patients after ASA was 99.6 (95%CI: 99.1–100.0) %, 98.6 (95%CI: 97.5–99.7) %, 97.9 (95%CI: 96.0–99.7) % at 1-, 5-, 10-year follow-up, respectively (Fig. 3).

Causes of long-term death were as follows: stroke (12/65 patients, 18 %); myocardial infarction (4/65 cases, 6 %); heart failure (9/65 cases, 14 %); sudden death (8/65 cases, 12 %), cancer (6/65 patients, 9 %); COVID-19 (5/65 cases, 8 %); trauma (2/65 cases, 3 %); unknown causes (19/65 cases, 29 %).

Multivariable analysis (Cox regression) identified 3 independent predictors of long-term death: age (HR 1.04, 95%CI: 1.02–1.06), IVS (HR 1.08, 95%CI: 1.02–1.18), resting peak gradient in the follow-up (HR 1.02, 95%CI: 1.00–1.13). C-statistic was 0.689 (See Table 4).

**Table 4:** Uni- and multivariate analysis.

Variable	Univariate				Multivariate			Multivariate final		
	**N**	**HR**	**95 % CI**	*p***-value**	**HR**	**95 % CI**	*p***-value**	**HR**	**95 % CI**	*p***-value**
**Female**	597	0.85	0.52, 1.39	0.5						
**Body-mass index**	597	1.02	0.97, 1.07	0.5						
**Age**	597	1.04	1.02, 1.06	<0.001	1.04	1.02, 1.07	<0.001	1.04	1.02, 1.06	<0.001
**Family history of sudden cardiac death**	597	0.83	0.38, 1.83	0.6						
**Pacemaker at baseline**	597	0.93	0.13, 6.74	>0.9						
**NYHA class at baseline**	597	1.34	0.93, 1.91	0.11						
**Coronary artery disease**	597	1.73	0.94, 3.19	0.1						
**Chronic obstructive pulmonary disease**	597	1.34	0.77, 2.34	0.3						
**Diabetes mellitus**	597	1.39	0.63, 3.04	0.4						
**Syncopes**	597	0.58	0.31, 1.09	0.075	0.6	0.32, 1.14	0.1			
**Arterial hypertension**	597	1.56	0.91, 2.70	0.1						
**Atrial fibrillation**	597	1.29	0.75, 2.20	0.4						
**Pacemaker at discharge**	597	1.17	0.51, 2.72	0.7						
**Left atrial diameter at baseline**	597	1.01	0.96, 1.06	0.7						
**Left ventricular end-diastolic diameter at baseline**	597	0.99	0.95, 1.03	0.6						
**Left ventricular ejection fraction at baseline**	597	0.99	0.96, 1.03	0.7						
**Interventricular septal thickness at baseline**	597	1.08	1.01, 1.16	0.027	1.09	1.01, 1.18	0.021	1.1	1.02, 1.18	0.015
**Septal reduction**	597	0.94	0.87, 1.02	0.2						
**Resting peak gradient at baseline**	597	1	1.00, 1.01	0.5						
**Provocable peak gradient at baseline**	597	1	0.99, 1.01	>0.9						
**Resting peak gradient in the follow-up**	597	1.02	1.01, 1.04	0.002	1.02	0.99, 1.04	0.2	1.02	1.00, 1.03	0.014
**Provocable peak gradient in the follow-up**	597	1.01	1.00, 1.02	0.011	1	0.98, 1.02	>0.9			
**Residual obstruction**	597	1.46	0.85, 2.52	0.2						

HR = Hazard Ratio, CI = Confidence Interval, NYHA = New York Heart Association.

The mean of the heart failure functional class improved from 2.3 ± 0.7 to 1.4 ± 0.5 (*p* < 0.001). The number of patients with III-IV NYHA functional class decreased from 42.1 % at baseline to 2 % in the follow-up.

During follow-up 72 patients underwent repeated ASA (12.0 %) and 14 patients underwent open heart surgery (myectomies - 12, myectomy plus mitral valve replacement – 2).

According to our definition, a residual obstruction after ASA was identified in 126 patients (21.1 %). Most of them (*n* = 74, 58.7 %) had NYHA class 1, 36.5 % (*n* = 46) patients were in NYHA class 2. Only 5 patients still suffered from the heart failure NYHA class 3.

### 4.5 Secondary analysis

In our cohort, we observed 72 patients who underwent repeated ASA during follow-up. We performed secondary analysis comparing outcomes in oHCM patients after single ASA versus repeated ASA. Baseline characteristics are presented in Supplementary Table 1. Initial imbalance between groups under study were adjusted using the inverse probability treatment weighting (IPTW) technique. A visual balance assessment was performed via “love plot” (Supplementary Fig. 3).

In unadjusted groups (Unbalanced cohort) we observed the higher NYHA class at 30-days follow-up (1.24 ± 0.46 vs 1.38 ± 0.54, *p* = 0.022), but the NYHA class in the follow-up was not statistically different. After adjustment (Weighted cohort) we found the lower permanent pacemaker implantations in patients at 30-days of follow-up after repeated ASA (2.8 %), comparing to those after single ASA (12 %, *p* = 0.024). Other predefined outcomes (30-day mortality, NYHA class at 30 days, NYHA class during follow-up, residual obstruction, LVOT obstruction during follow-up, myectomy after ASA sessions) did not show statistically significant differences between the groups after adjustment using inverse probability of treatment weighting (IPTW). See Supplementary Table 2.

The long-term survival rates in patients after repeated ASA were similar to those after single ASA (adjusted HR 0.65, 95%CI: 0.31–1.36, *p* = 0.254).

In a sensitivity analysis we excluded (filtered) patients who underwent myectomy in the follow-up and repeated survival analysis. In this filtered cohort the long-term survival rates in patients after repeated ASA were similar to those after single ASA (adjusted HR 0.57, 95%CI: 0.27–1.38, *p* = 0.138). See Supplementary Figs. 1–2.

## 5 Discussion

HCM is a widespread disease with an incidence of 1:200 in the general population. It is assumed that 15 million people are affected globally.^10^ Surgical treatment was introduced in the US in the early 60s and then got highly developed in North America.^11^ Ulrich Sigwart developed a less invasive technique (ASA) in the 90s which became popular in Europe.^12^ However, for a long time in Russia, there was no well-structured surgical HCM program and in the early 2000s, two independent groups of interventionists started with ASA.^13^ At this moment in our country there is a surgical group with 500+ myectomies experience with impressive results but taking into account the huge territory of the Russian Federation HCM population still seems undertreated.^14^ Data from the NIS registry demonstrated that ASA seems more reproducible than surgical myectomy.^15^ Our report demonstrates the safety of ASA in two leading Russian centers**.**

However, the ASA technique has not changed a lot in the last decade. But the increasing knowledge of procedural effects and ASA's inherited advances and limitations leads to better patient's selection and improvement in results.

This registry expands knowledge of ASA outcomes in developing countries, particularly in Russian Federation. Outcomes of ASA in European or US patients were studied very well previously.^1^^,^^2^ However, population of developing countries has a different socio-economic status comparing to western countries.

### 5.1 Safety endpoint

To the best of our knowledge, this registry demonstrates the lowest hospital mortality (0.7 %) and the lowest need in permanent pacemaker implantations (7 %). The largest registries (European and North American) demonstrate an 8.9–12 % frequency of permanent pacemaker implantations after ASA.^1^^,^^2^ Real-world data from the U.S. Nationwide Inpatient Database showed the 11.9 % PPM implantations after ASA.^15^ In this registry, such complications as tamponade or ventricular septum defects after ASA were not observed.

We would like to state that ASA results in our cohort of HCM patients are seem close to the AHA/ACC targets for invasive septal reduction therapies outcomes.^5^

### 5.2 Efficiency based on the operative threshold

However, the residual obstruction is an underreported issue in papers with ASA. In most reports, a freedom from reoperation is the most commonly used measure.^13^ Based on the operative threshold (provoked PG ≥ 50 mmHg) in our cohort residual obstruction after ASA was identified in 126 patients (21.1 %).

It may be explained by the following.

(i) Practically, this experience includes patients close to all-comers cohort. We did not exclude oHCM patients with long leaflets, mitral abnormalities, mid-cavity obstruction, difficult septal anatomy (thin <17 mm, or thick ≥30 mm).
(ii) Mean ethanol dose in this study was 1.6 ± 0.9 ml. In Veselka's study there was the following ethanol dose: 0.4–11 mL, the median was 2.0 ml. Batzner A et al reported an experience of 900+ ASA procedures with 2.1 ± 0.4 ml.

However, in our registry the lower dose was used. We consider that this good safety profile reported in the study (low mortality of 0.7 %, relatively low pacemaker rates of 7 %) was at the cost of the relatively higher residual obstruction rates comparing to previous researches.^1^^,^^2^^,^^16^

### 5.3 Repeated ASA

Repeated ASA was performed due to insufficient gradient reduction after the first attempt and persistent symptoms or as a staged procedure in cases of multiple perforators pattern as it proposed by Seggewiss H.^16^ We performed secondary analysis to assess safety and efficiency of repeated ASA.

In our study 72 patients underwent repeated ASA. However, at the moment data about oHCM patients after repeated ASA are scarce. Previous reports from largest ASA series included 164 (18.1 %) patients after repeated ASA from Hubert Seggewiss group, and 145 (10 %) oHCM patients from Euro-ASA registry.^16^^,^^17^ A study from Veselka J et al was focused on outcomes of so called “repeated septal reduction therapy” which included both of ASA and myectomy.^17^

We performed our secondary analysis in two ways: (1) we analyzed outcomes comparing single ASA versus repeated ASA; (2) we compared survival in single ASA versus repeated ASA (myectomy patients were excluded) as a sensitivity analysis. See Fig. 1.

We studied the following outcomes: (i) 30-day mortality, (ii) 30-day permanent pacemaker implantations, (iii) residual obstruction, (iv) final maximal left ventricular outflow tract gradient, (v) long-term mortality, (vi) final heart failure functional class, (vii) myectomy cases after ASA sessions.

In unbalanced cohort we observed the higher heart failure functional class in patients after repeated ASA (1.24 ± 0.46 vs 1.38 ± 0.54, *p* = 0.022), but after IPTW-adjustment groups were not statistically different in terms of the heart failure severity (*p* = 0.093). See Supplementary Table 2.

Interestingly, in a weighted cohort we found the lower permanent pacemaker implantations in patients after repeated ASA (2.8 %), comparing to those after single ASA (12 %, *p* = 0.024). However, this phenomenon may be explained by the following.

1. Theoretically, a muscular bulge in the LVOT may be perfused by just a single perforator or by multiple perforators. Performing ASA in cases of the single perforator we are damaging the larger volume of basal septum comparing to patients with the multiple perforators pattern underwent staged ablation (1 perforator per procedure).
2. However, patients with the multiple perforators pattern could suffer from more extensive forms of HCM (not only basal hypertrophy), including neutral septum, reverse curve or apical hypertrophy. Unfortunately, in our study these data were not collected.

Other outcomes which we initially specified were not statistically different between groups after IPTW-adjustment.

Weighted survival analysis didn't reveal a difference between groups with single versus repeated ASA (adjusted HR 0.65, 95%CI: 0.31–1.36, *p* = 0.254). See Supplementary Fig. 1. This finding is consistent with previous study from Veselka J., which compared single ASA (*n* = 1240) versus repeated septal reduction therapy (both repeated ASA and myectomy, *n* = 145), *p* = 0.165 ^17^.

Both groups of patients in the follow-up underwent the statistically similar number of myectomies (4 % vs 1 %, *p* = 0.3). To ensure in our findings and decrease an influence of myectomies on final results, we decided to filter patients who underwent myectomies in the follow-up and repeated survival analysis. Weighted survival in this filtered cohort of oHCM patients were not different between groups under study (adjusted HR 0.57, 95%CI: 0.27–1.2, *p* = 0.138). See Supplementary Fig. 2.

### 5.4 Septal reduction in ASA and SM

This paper demonstrates that the mean septal reduction in our cohort was 4.4 ± 2.8 mm. The largest Euro-ASA registry showed the mean septal reduction of 5 mm.^1^ What does it mean for practice? Well-recognized surgical myectomy teams showed that the mean septal reduction ranges from 8 to 10 mm.^14^^,^^18^^,^^19^ However, this fact illustrates the fundamental difference between ASA and myectomy in terms of procedural effects. Does the lower septal reduction lead to worse outcomes? This question is still open. However, future studies will clarify this issue.

## 6 Causes of death and its explanation

In our study 38 % patients died from cardiovascular reasons (stroke, myocardial infarction, heart failure). It seems a bit higher frequency than in previous studies. Veselka J et al reported 21 % of deaths from cardiovascular reasons in the Euro-ASA registry.^1^ Batzner A et al showed 20 % cardiovascular death occurrences.^16^

It seems that in our HCM population, cardiovascular reasons of death were predominant. However, it might be explained by the different socio-economical status of Russian citizens comparing to western countries. According to WHO reports, Russian population had the higher prevalence of myocardial infarction and stroke per 10 000 citizens comparing to that in western countries.^20^

## 7 Predictors of death

Multivariate analysis revealed 3 predictors of all-cause death in the follow-up: (i) age, (ii) IVS thickness at baseline, and (iii) resting peak gradient in the follow-up. However, our model was very close to the ASA-SCARRE formula created by Veselka J el al.^23^ It is worth noting that some studies showed lower survival and a higher residual obstruction rate after ASA in oHCM patients who had preexisting myocardial fibrosis.^21^^,^^22^ Unfortunately, we were not able to analyze such a variable as myocardial fibrosis due to high missingness.

## 8 Limitations

This study is observational, retrospective. It has a potential risk of selection bias that should be considered before generalization of the results. Data were collected from predominantly two HCM center and from three ASA operators. There were some operator-specific differences in the strategy of the ethanol dose selection. We analyzed data from different operators together assuming all steps of ASA procedure were identical and the dose selection strategy was not meaningful for outcomes in this study. Besides, our main analysis was descriptive in its nature. Secondary analysis compared outcomes in patients after single versus repeated ASA. However, secondary analysis was underpowered for the long-term survival endpoint.

We consider that in our study all patients were followed. In cases when we lost contact with patients or their relatives, we used data from Insurance Fund to know a current state (is patient alive or died, date of death). A mortality count did not include cases of ICD discharges.

## 9 Conclusions

In our registry, alcohol septal ablation in patients with obstructive hypertrophic cardiomyopathy was safe in the short- and long-term follow-up. Outcomes of patients underwent repeated ASA were non-inferior to those after single ASA.

Our study showed that a lower dose of ASA (1.6 ± 0.9 ml) was effective in reducing outflow gradients, leading to low rates of in-hospital mortality and pacemaker implantation. However, this benefit was tempered by a reoperation need of 12.1 % and residual obstruction in 21.1 % of cases. Nevertheless, repeated ASA for residual obstruction had low complication rates and high effectiveness. This underscores the potential for a sequential approach to further refine this procedure.

‘What is Already Known?’

• Alcohol septal ablation is a well-established procedure with over than 20 years history
• The largest experience of ASA collected in Europe and USA. It was demonstrated that ASA is safe and efficient procedure

‘What this Study Adds?’

• ASA in Russia was also safe and efficient
• The low dose ASA associated with a brilliant safety profile at the cost of its efficiency
• Repeated ASA demonstrated similar safety profile as a single ASA
• Patient after repeated ASA had the similar long-term survival comparing to those after single ASA
• In our cohort repeated ASA was associated with the lower need in permanent pacemakers in 30-day follow-up

## Funding

None.

## Declaration of competing interest

The authors declare that they have no known competing financial interests or personal relationships that could have appeared to influence the work reported in this paper.
