# Supplementary material for: National experience of alcohol septal ablation in patients with obstructive hypertrophic cardiomyopathy: A long-term multicenter retrospective study
Source: Indian Heart J. 2024 Nov 22;76(6):390–7. doi: 10.1016/j.ihj.2024.11.248 (PMC11705596; doi:10.1016/j.ihj.2024.11.248)
Supplement: Multimedia component 1 [file mmc1.docx]

**Supplementary material**

| **Supplementary table 1. Baseline characteristics of oHCM patients after single ASA versus repeated ASA** | | | | | | |
| --- | --- | --- | --- | --- | --- | --- |
|  | ****Unbalanced**** | | | ****Weighted**** | | |
| **Variable** | **Single ASA^1^, N = 525** | **Repeated ASA^1^, N = 72** | **p-value^2^** | **Single ASA^1^, N = 73** | **Repeated ASA^1^, N = 72** | **p-value^3^** |
| **female** |  |  | 0.8 |  |  | 0.9 |
| 0 | 238 (45%) | 31 (43%) |  | 32 (44%) | 31(43%) |  |
| 1 | 287 (55%) | 41 (57%) |  | 41 (56%) | 41 (57%) |  |
| **Body-mass index, kg/m2** | 29.6 (4.9) | 30.1 (4.8) | 0.4 | 30.0 (4.9) | 30.1 (4.8) | 0.8 |
| **Age, years** | 56 (14) | 55 (16) | 0.5 | 55 (15) | 55 (16) | 0.6 |
| **Pacemaker** |  |  | 0.14 |  |  | 0.7 |
| 0 | 519 (99%) | 69 (96%) |  | 69 (95%) | 69 (96%) |  |
| 1 | 6 (1%) | 3 (4%) |  | 4 (5.3%) | 3 (4.2%) |  |
| **NYHA class at baseline** |  |  | 0.013 |  |  | 0.3 |
| 1 | 60 (11%) | 1 (1.2%) |  | 4 (5.6%) | 1 (1.4%) |  |
| 2 | 252 (48%) | 33 (46%) |  | 28 (39%) | 33 (46%) |  |
| 3 | 199 (38%) | 36 (50%) |  | 36 (50%) | 36 (50%) |  |
| 4 | 14 (3%) | 2 (2.8%) |  | 4 (5.6%) | 2 (2.8%) |  |
| **Coronary artery disease** |  |  | >0.9 |  |  | 0.9 |
| 0 | 448 (85%) | 62 (86%) |  | 63 (87%) | 62 (86%) |  |
| 1 | 77 (15%) | 10 (14%) |  | 10 (13%) | 10 (14%) |  |
| **Chronic obstructive pulmonary disease** |  |  | 0.2 |  |  | 0.2 |
| 0 | 396 (75%) | 60 (83%) |  | 55 (76%) | 60 (83%) |  |
| 1 | 129 (25%) | 12 (17%) |  | 17 (24%) | 12 (17%) |  |
| **Diabetes mellitus** |  |  | 0.14 |  |  | >0.9 |
| 0 | 478 (91%) | 61 (85%) |  | 61 (85%) | 61 (85%) |  |
| 1 | 47 (9%) | 11 (15%) |  | 11 (15%) | 11 (15%) |  |
| **Syncopes** |  |  | 0.8 |  |  | 0.7 |
| 0 | 354 (67%) | 47 (65%) |  | 49 (68%) | 47 (65%) |  |
| 1 | 171 (33%) | 25 (35%) |  | 24 (32%) | 25 (35%) |  |
| **Arterial hypertension** |  |  | 0.2 |  |  | >0.9 |
| 0 | 164 (31%) | 28 (39%) |  | 29 (39%) | 28 (39%) |  |
| 1 | 361 (69%) | 44 (61%) |  | 44 (61%) | 44 (61%) |  |
| **Family history of sudden cardiac death** |  |  | 0.3 |  |  | 0.063 |
| 0 | 452 (86%) | 58 (81%) |  | 64 (88%) | 58 (81%) |  |
| 1 | 73 (14%) | 14 (19%) |  | 8 (12%) | 14 (19%) |  |
| **Atrial fibrillation** |  |  | 0.6 |  |  | 0.8 |
| 0 | 419 (80%) | 55 (76%) |  | 55 (75%) | 55 (76%) |  |
| 1 | 106 (20%) | 17 (24%) |  | 18 (25%) | 17 (24%) |  |
| **Ventricular tachycardia** |  |  | 0.8 |  |  | 0.4 |
| no | 513 (97%) | 71 (99%) |  | 70 (96%) | 71 (99%) |  |
| non-sustain VT | 9 (2%) | 1 (1%) |  | 2 (2.3%) | 1 (1.4%) |  |
| sustain VT | 3 (1%) | 0 (0%) |  | 1 (2.0%) | 0 (0%) |  |
| **Left atrial diameter at baseline, mm** | 42.7 (4.8) | 44.2 (4.3) | 0.009 | 44.3 (5.5) | 44.2 (4.3) | 0.5 |
| **Left ventricular end-diastolic diameter at baseline, mm** | 46.3 (6.1) | 46.5 (4.9) | 0.8 | 46.4 (6.5) | 46.5 (4.9) | 0.3 |
| **Left ventricular ejection fraction at baseline, %** | 67 (7) | 68 (7) | 0.4 | 68 (7) | 68 (7) | 0.6 |
| **Interventricular septal thickness at baseline, mm** | 21.2 (3.0) | 21.2 (3.1) | >0.9 | 21.2 (3.2) | 21.2 (3.1) | 0.9 |
| **Left ventricular outflow tract obstruction at baseline, mmHg** | 103 (30) | 106 (33) | 0.4 | 106 (32) | 106 (33) | >0.9 |
| ^1^n (%); Mean (SD) | | | | | | |
| ^2^Pearson's Chi-squared test; Two Sample t-test; Wilcoxon rank sum test | | | | | | |
| ^3^chi-squared test with Rao & Scott's second-order correction; Wilcoxon rank-sum test for complex survey samples | | | | | | |
| Abbreviations: oHCM - obstructive hypertrophic cardiomyopathy, ASA - alcohol septal ablation, VT - ventricular tachycardia | | | | | | |

| Supplementary table 2. Outcomes in oHCM patients after single versus repeated ASA | | | | | | |
| --- | --- | --- | --- | --- | --- | --- |
|  | **Unbalanced** | | | **Weighted** | | |
| Variable | **Single ASA^1^,**  **N = 525** | **Repeated ASA^1^,**  **N = 72** | **p-value^2^** | **Single ASA^1^,**  **N = 73** | **Repeated ASA^1^,**  **N = 72** | **p-value^3^** |
| 30-day mortality | 3 (0.6%) | 1 (1.4%) | >0.9 | 0 (0%) | 1 (1%) | 0.4 |
| Pacemaker at 30-days |  |  | 0.2 |  |  | **0.024** |
| no | 485 (92%) | 70 (97%) |  | 64 (88%) | 70 (97%) |  |
| yes | 40 (7.6%) | 2 (2.8%) |  | 9 (12%) | 2 (3%) |  |
| NYHA class at 30-days | 1.24 (0.46) | 1.38 (0.54) | **0.022** | 1.26 (0.47) | 1.38 (0.54) | 0.093 |
| NYHA class in the follow-up | 1.31 (0.51) | 1.32 (0.53) | >0.9 | 1.36 (0.53) | 1.32 (0.53) | 0.4 |
| Residual obstruction (at last follow-up visit), % |  |  | >0.9 |  |  | 0.8 |
| no | 415 (79%) | 56 (78%) |  | 56 (77%) | 56 (78%) |  |
| yes | 110 (21%) | 16 (22%) |  | 17 (23%) | 16 (22%) |  |
| Left ventricular outflow tract obstruction in the follow-up, mmHg | 17 (12) | 18 (10) | 0.9 | 19 (14) | 18 (10) | 0.9 |
| Myectomy after ASA sessions  no  yes | 512 (97.5%)  13 (2.5%) | 71 (98.6%)  1 (1.4%) | 0.876 | 70 (96%)  3 (4%) | 71 (99%)  1 (1%) | 0.3 |
| ^1^n (%); Mean (SD) | | | | | | |
| ^2^Pearson's Chi-squared test; Wilcoxon rank sum test; Two Sample t-test | | | | | | |
| ^3^chi-squared test with Rao & Scott's second-order correction; Wilcoxon rank-sum test for complex survey samples | | | | | | |
| Abbreviation: oHCM - obstructive hypertrophic cardiomyopathy, ASA - alcohol septal ablation, NYHA - New York Heart Association | | | | | | |

**
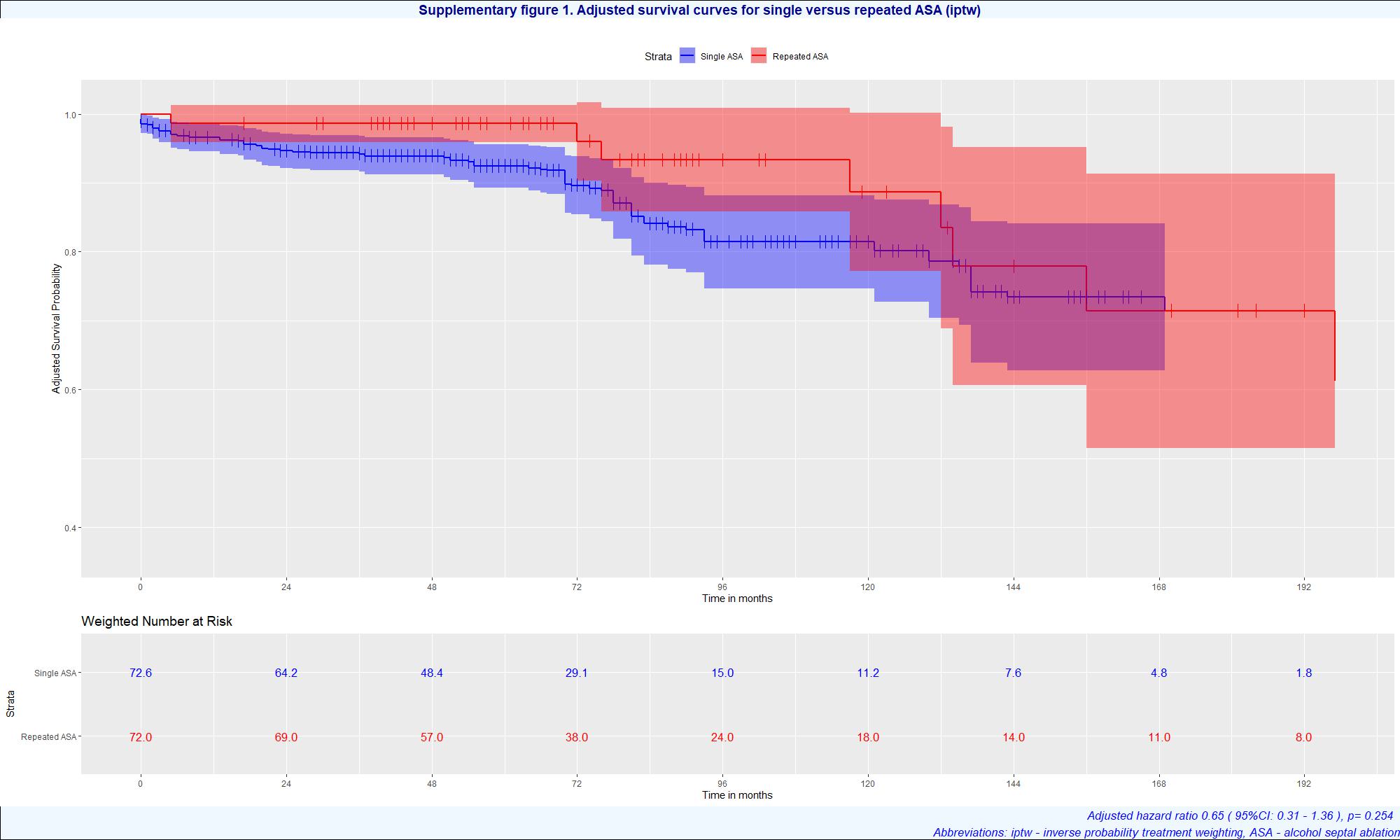
**

**
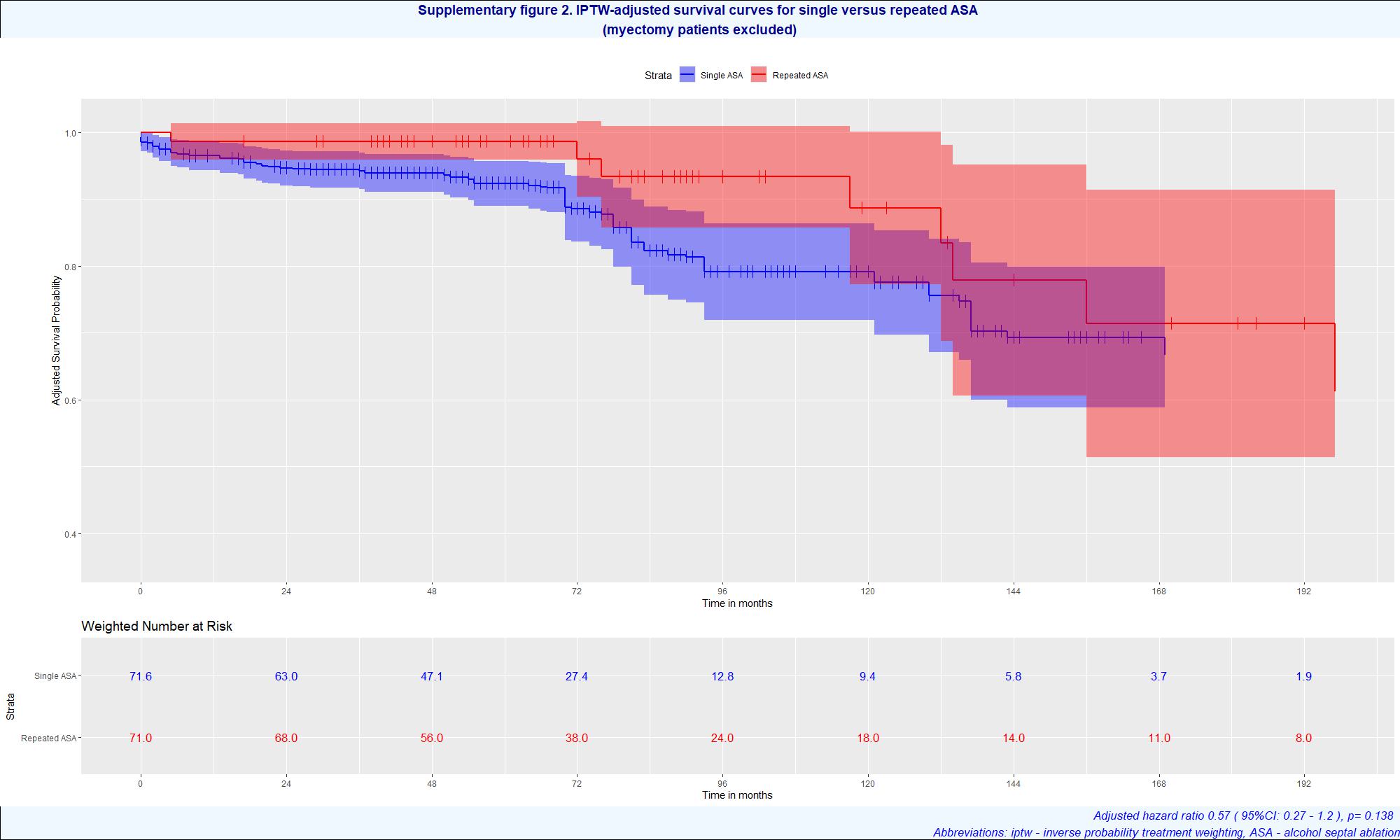
**

**
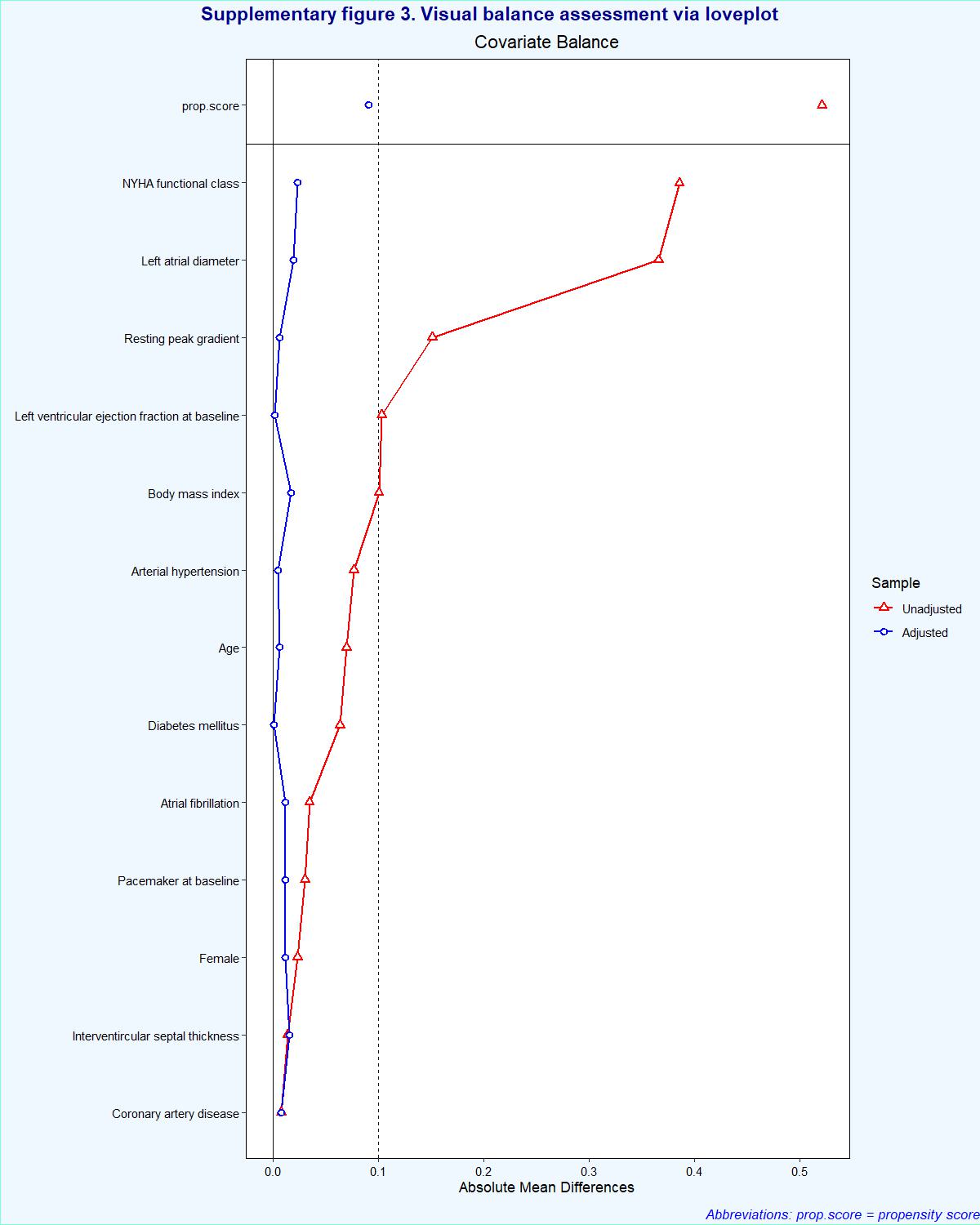
**
